# Prevalence of Oral Habits among Eleven to Thirteen Years Old Children in Jaipur

**DOI:** 10.5005/jp-journals-10005-1314

**Published:** 2015-09-11

**Authors:** Shantanu Sharma, Ayushi Bansal, Kirti Asopa

**Affiliations:** Postgraduate, Department of Orthodontics, Mahatma Gandhi Dental College, Jaipur, Rajasthan, India; Postgraduate, Department of Pedodontics, Mahatma Gandhi Dental College, Jaipur, Rajasthan, India; Assistant Professor, Department of Pedodontics, Mahatma Gandhi Dental College, Jaipur, Rajasthan, India

**Keywords:** Children, Oral habit, Tongue thrusting.

## Abstract

**Aim:** Oral habits that are prevalent well beyond the normal age frequently result in facial deformity and malocclusions. The aim of the present study was to know the prevalence of oral habits in 11 to 13 years old children of Jaipur city.

**Methodology:** The study included 1,000 children of age 11 to 13 years, belonging to different government and private schools of Jaipur city who were screened for any deleterious habits at their school site. The statistical analysis was done using Chi-square test.

**Results:** The result showed that 18% children had a habit of tongue thrusting, 17% mouth breathing and 3% nail biting. Sex-wise prevalence showed 18% females had oral habits and 20% of male had oral habit.

**Conclusion:** The distribution of children aged 11 to 13 years having oral habits was evaluated with tongue thrusting being most prevalent and exhibiting minimal sexual predilection.

**How to cite this article:** Sharma S, Bansal A, Asopa K. Prevalence of Oral Habits among Eleven to Thirteen Years Old Children in Jaipur. Int J Clin Pediatr Dent 2015;8(3):208-210.

## INTRODUCTION

A habit is defined as an automatic repetitive action as a result of complex natural process involving muscle contraction. Habit may be a normal or an abnormal habit. Normal habits serve as a constructive role in growth pattern while abnormal habits may lead to disturbances in normal growth pattern.

Oral habits are learned patterns of muscle contraction and have a very complex nature. Persistent oral habits beyond the normal age have been considered as an important factor which may lead to the malocclusion. Anterior open bite, posterior crossbite, incisor protrusion, lip incompetence distal step molar relation are some of the common negative consequences caused by the prolonged oral habits. The extent of these disturbances varies from child to child depending on their actual skeletal and dental relationship and their inherent actual habit.

The prevalence of oral habits in high school girls and primary school students have been reported to be 87.9 and 30% respectively (Yassaei et al 2004). Quashie-Williams et al (2007)^1^ found that 34.1% of children were having deleterious oral habit.

According to many researchers finger sucking and nail biting are the most frequent ones present during childhood. Digit sucking is more common among young children while nail biting in older children. This finding may be attributed to the fact that digit sucking is closely related to the child’s psychoemotional maturity and considered as normal phenomenon in the first 2 years with a reported prevalence of 20 to 30%. From the age of 4 to 7 years, finger sucking has been reported to have prevalence between 5 and 17% in different populations. Other oral habits, such as lip biting, tongue thrusting, lip sucking, bruxism are sometimes observed in children but to a lesser extent. Their lower prevalence rates could be due to difficulty to notice such oral habits.^2^

These studies are done to plan the prevention and eradication of these oral habits so as to reduce the occurrence of malocclusion, further contributing in the rise of national level of oral health.

The present study is conducted with aim of knowing the prevalence of oral habits in 11 to 13 years old children of Jaipur city, so that their deleterious effects can be prevented and eradicated.

## MATERIALS AND METHODS

The study included 1000 children of age 11 to 13 years, belonging to different government and private schools of Jaipur city. Out of 1,000 children, 540 were males and 460 were females. The result and study model has been shown in Table 1.

Children were examined on an upright chair using torch light, mouth mirror and straight probe. Prevalence rates of different oral habits studied were calculated. Statistical package for the social sciences (SPSS) version 15.0 statistical package was used. Chi-square test was done to compare the prevalence of oral habits among different sexes, the value of p < 0.05 was regarded as significant.

## RESULTS

Prevalence of oral habits in males and females is shown in Table 2. Age-wise sample distribution is shown in Table 3. The result showed that 18% children had a habit of tongue thrusting, 17% mouth breathing and 3% nail biting.

Sex-wise prevalence showed 18% females had oral habits and 20% of male had oral habit. Considerable differences between male and female when nail biting was found.

## DISCUSSION

Present study was conducted with aim of knowing the prevalence of oral habits in 11 to 13 years old children of Jaipur city, so that deleterious effects of same can be prevented.

Documented in studies from different parts of the world regarding child’s oral habit have well confirmed that habits affect the development of occlusion hence, play a pivotal role in facial appearance of the child.^2-6^

Further, the children examined had oral habit of some or the other kind. This finding is in agreement with the results of Quashie-Williams,^1^ who found 34.1% of the children examined presented with an oral habit. In contrast to this observation low prevalence of oral habits (29.7 and 25.5%) was reported by Shetty et al (1998)^7^ and Kharbanda et al (2003)^8^ who studied prevalence of oral habits in south and north Indian children respectively. Further, Guaba et al^9^ reported that only 3% of children demonstrated oral habits, which is very much in disagreement with our findings. But higher prevalence (50%) of oral habits had been reported by Bandung et al^10^ who did a study on children of age 6 to 12 years. Tongue thrusting and mouth breathing were the most prevalent oral habits in the present study sample. Our findings do agree with the observation of Guaba et al^9^ and Kharbanda et al.^8^ Whereas digit sucking was the most frequently occurring oral habits seen in 50% of the children in the reports of Quashie-Williams et al^1^ present study revealed that tongue thrusting habit was prevalent in 18% of the children; same is supported by the findings of Kharbanda et al^8^ who reported 18.1% of children with tongue thrusting habit. However, our findings differed with the findings of Shetty and Munshi^7^ who found a comparatively low prevalence (3.02%) of tongue thrust among Mangalore children in the age range of 3 to 16 years.

**Table 1:** **Table 1:** Sample size distribution

*Age*		*Males*		*Females*		*Total*	
11		250		210		460	
12		180		140		320	
13		110		110		220	
Total		540		460		1000	

**Table 2:** **Table 2:** Sex-wise prevalence of oral habit

*Sl. no.*		*Type of habit*		*Males* * (n = 540)*		*Females* * (n = 460)*		*p-value*	
1.		Digit sucking		0 (0%)		0 (0%)		―	
2.		Tongue thrusting		100 (10%)		80 (8%)		> 0.05 (NS)	
3.		Mouth breathing		90 (9%)		80 (8%)		> 0.05 (NS)	
4.		Bruxism		0 (0%)		0 (0%)		―	
5.		Lip biting		0 (0%)		0 (0%)		―	
6.		Nail biting		10 (1%)		20 (2%)		< 0.05 (S)	

NS: Not significant; S: Significant

**Table 3:** **Table 3:** Age-wise prevalence of oral habits

*Age (years)*		*N*		*Prevalence (%)*	
11		460		100 (10)	
12		320		120 (12)	
13		220		80 (8)	

Mouth breathing habit was the second most prevalent habit in our study with the incidence rate of 17%. This incidence was higher when compared to the findings of the previous studies.^7,8^ Amr Abou-Ei-Ezz et al^11^ in their study on prevalence of mouth breathing habit and its probability as etiological factor of malocclusion have concluded that malocclusion is highly associated with habits existence and this relationship is statistically highly significant (p < 0.001).

Nail biting habit was seen only in 3% of children and it was the most prevalent habit after tongue thrusting and mouth breathing which contrasted the study by Shetty and Munshi,^7^ which reported occurrence of 12.7% children with nail biting. It was higher to what was found in our study.

Other habits like thumb sucking, bruxism and lip biting was absent in the present group.

There was difference in prevalence of oral habits according to the age. Oral habits were found to be more prevalent in 21 years old children (12%). And least was found in 13 years olds (8%).^12,13^

On comparing the male and female prevalence of oral habits, difference was seen in nail biting habit with higher prevalence in females.^14^ The reason behind the gender-wise difference in the occurrence of oral habits is due to the fact that oral habits in boys are more persistent for longer period than girls because boys tend to openly fight against family’s or surrounding society’s rules than girls, including when they are told to stop practicing oral habits.

The most prevalent habit in our study was tongue thrusting and the reason attributed for this was the constant changeover of teeth in mixed dentition often leading to open spaces, thereby prompting a habit of tongue thrusting. However, a more accurate measure of oral habits in children would be possible with wider age groups.
